# Long-Circulating and Fusogenic Liposomes Loaded with Paclitaxel and Doxorubicin: Effect of Excipient, Freezing, and Freeze-Drying on Quality Attributes

**DOI:** 10.3390/pharmaceutics15010086

**Published:** 2022-12-27

**Authors:** Marjorie Roque, Danilo Geraldes, Caroline da Silva, Mônica Oliveira, Laura Nascimento

**Affiliations:** 1 Department of Pharmaceutical Products, Faculty of Pharmacy, Universidade Federal de Minas Gerais, Belo Horizonte 31270-901, Minas Gerais, Brazil; 2 Faculty of Pharmaceutical Sciences, Universidade de Campinas, Campinas 13083-970, São Paulo, Brazil

**Keywords:** freeze-drying, liposomes, doxorubicin, paclitaxel, co-encapsulation, breast cancer

## Abstract

Liposomes can increase plasma half-life, enhance targeting, and diminish the side-effects of loaded drugs. On the downside, physical and chemical instabilities of dispersions often result in a reduced lifespan, which limits their availability on the market. Solid formulations obtained by freeze-drying can immobilize vesicles and provide extended shelf life. For both processes, the choice of excipients and process parameters are crucial to protect the carrier layers against tension caused by freezing and/or dehydration. The aim of this work is to evaluate the influence of freezing and drying parameters, besides excipient choice, to obtain solid long-circulating and fusogenic liposomes (LCFL-PTX/DXR) co-encapsulating paclitaxel (PTX) and doxorubicin (DXR) at a synergistic ratio (1:10). Methods: LCFL-PTX/DXR was evaluated by freeze-drying microscopy (glass transition, Tg’), differential scanning calorimetry (collapse temperature, Tc), freeze-thawing and freeze-drying processes. Freeze-dried samples were evaluated by thermogravimetry (residual moisture) and the resuspended liposomes were characterized in terms of size, polydispersity index (PI), zeta potential (ZP), and drug content. Liposomes morphology was evaluated by cryomicroscopy. Results: Trehalose protected PTX cargo upon freeze-thawing and more than 80% of the original DXR retention. The formulations with trehalose resulted in a cake with 5–7% of moisture content (200–240 nm); 44–60% of PTX retention, and 25–35% of DXR retention, with the variations caused by cryoprotector concentration and process changes. Conclusions: Trehalose protected liposome integrity, maintaining PTX retention and most of DXR upon freeze-thawing. Freeze-drying reduced the retention of both drugs inside all liposomes, whereas formulation with trehalose presented minor losses. Therefore, this frozen formulation is an alternative product option, with no need for manipulation before use.

## 1. Introduction

Liposomes as carrier systems bring good drug loading, adjustable physicochemical and biological properties, and biocompatibility due to their resemblance to biological membranes. The liposomal products show significant enhancement of therapies, with a highlight on anticancer treatment with doxorubicin (DRX). In particular, our research group recently showed that a combination of DRX and paclitaxel (PTX) loaded liposomes not only inhibits breast tumors but also abrogates the cardiac toxicity induced by the free drugs in mice [1].

On the downside, liposomes can suffer lipid oxidation, hydrolysis, drug leakage, and aggregation/fusion of vesicles during their shelf life. The addition of saturated lipids and antioxidants may prevent chemical degradation, whereas functionalized or charged vesicles enhance colloidal stability [1,2,3,4,5]. Explicitly, long-circulating liposomes generally contain polyethyleneglycol (PEG) moieties that prevent fusion/aggregation between particles, and consequently, colloidal stability; in physiological terms, they diminish phagocyte uptake and increase plasma half-life. However, even formulation design may not be sufficient: in the case of PEG addition, this strategy maintained particle size and distribution but did not prevent DRX leakage from PEG-containing liposomes over time, [6]. 

The reported phenomena occur in the aqueous liquid medium. Therefore, a way to circumvent these problems of instability is to dry or freeze the formulations. Both strategies immobilize solutes at the end, but vesicles can be damaged along the process [2,3,4,5,7]. The freezing separates most of the solvent from liposomes and excipients, resulting in the formation of ice crystals [3,5]. Since water molecules interacting with polar moieties of liposome lipids contribute to spatial membrane distribution, dehydration may favor vesicle fusion/aggregation and modify the packing density and transition temperature of lipids [2,8,9].

Among the drying modes, freeze-drying is a frequently used one for liposomes. It can provide shelf-life extension and storage at higher temperatures than liquid forms. Since it is expensive and time-consuming, it needs to be as fast as possible, reproducible, scalable, and robust. Thus, all steps must be carefully evaluated [10]. A typical freeze-drying process consists of three main phases: freezing, primary drying, and secondary drying [3,11,12]. The kinetics of ice nucleation and crystal growth in freezing determine the final properties of the lyophilized product, since the ice morphology is directly correlated with the sublimation rate in primary and secondary drying [13,14].

During primary drying, the ice crystals from the freezing step sublime, corresponding to the free solvent portion [15,16]. The next step, secondary drying, aims to reduce moisture to an optimum level of product stability, since even after primary drying the product still contains 10–35% bound water [17,18,19,20]. For this purpose, the shelf temperature is increased to allow water desorption. All of these steps can be critical for the integrity of liposomes and the retention of encapsulated compounds [3,5]. The definitions of these freeze-drying technological parameters mainly focus on protecting lipid bilayers against damage caused by ice crystal growth during freezing, inhibition of vesicle melting/aggregation after dehydration, and prevention of a phase transition during rehydration [8]. Otherwise, the main consequences would be rupture, aggregation with a possible increase in vesicle size, and low drug encapsulation. The inclusion of cryoprotectants in the liposomal formulation is performed in order to minimize damage to the final product [21,22,23]. For this, the type and concentration of the cryoprotectant must be investigated [3,7,11]. 

Considering the above, in this work, we investigated the influence of different aspects of the freezing and freeze-drying process on quality attributes of liposomal formulations containing paclitaxel (PTX) and doxorubicin (DXR) (LCFL-PTX/DXR). Different cryoprotectants such as glucose, sucrose, and trehalose were tested at different concentrations, being them 2:1; 3:1, 5:1, and 9:1 *w*/*w* ratios in relation to the phospholipids. The cryoprotection and lyoprotection capacity on LCFL-PTX/DXR was evaluated by determining drug retention, size, polydispersity index, and zeta potential of the particles after freezing/thawing and lyophilization/rehydration. Variations in lyophilization cycles in relation to freezing and drying temperatures, vacuum, and time of each step were also verified. Lyophiles were investigated for residual moisture, particle size and distribution (NTA), collapse temperature (lyophilization microscopy), and their morphology (cryomicroscopy).

These formulations proved to be an efficient system for the experimental treatment of breast cancer, as demonstrated by the physicochemical characterization and biological effects observed in vitro and in vivo [1,24,25,26], but were not stable as liquid dispersions for an extended shelf-life period. We believe this work can shed light on solid formulations of antitumor drug-loaded long-circulating liposomes as a stability strategy, applicable to the studied drugs but also drugs with similar physicochemical attributes.

## 2. Materials and Methods

### 2.1. Material

1,2-Dioleoyl-sn-glycero-3-phosphoethanolamine (DOPE) and 1,2 distearoyl-sn-glycero-3-phosphoethanolamine-N-[amino(polyethyleneglycol) 2000 (DSPE-PEG2000) were supplied by Lipoid GmbH (Ludwigshafen, Germany). Cholesterol hemisuccinate (CHEMS), doxorubicin (DXR), [4-(2-Hydroxyethyl)-1-piperazinyl]-ethanesulfonic acid sodium salt, sodium chloride, sodium hydroxide, and Cremophor EL were obtained from Sigma-Aldrich Co. (St. Louis, MO, USA). Paclitaxel (PTX) was supplied by Quiral Quimica do Brasil S.A (Juiz de Fora, Brazil). Trehalose, glucose, and sucrose were purchased from VETEC Química Fina LTDA (Rio de Janeiro, Brazil). All other chemicals used in this study were of analytical grade. 

### 2.2. Preparation and Characterization of LCFL-PTX/DXR

#### 2.2.1. Liposome Preparation

LCFL-PTX/DXR were prepared by the lipid film hydration technique using a rotary evaporator Buchi Labortechnik AG CH-9233, model R-210, coupled to a V-700 vacuum pump of the same brand (Flawil, Switzerland). For this, chloroform aliquots of DOPE, CHEMS, and DSPE-PEG2000 (total lipid concentration of 10 mM, molar proportions of 5.7: 3.8: 0.5, respectively) and PTX (0.25 mg/mL) were transferred to a round bottom flask and submitted to evaporation in a water bath at 50 °C to remove the solvent. The lipid film obtained was then kept for 1 h under an atmosphere of chloroform for better dispersion of PTX in the lipids (known as chloroform annealing) [27].

An aliquot of 0.1 M NaOH solution was added to the film to promote the complete ionization of CHEMS molecules and the subsequent formation of a lamellar structure. The lipid film was hydrated with a solution of ammonium sulphate (300 mM, pH 7.4) preheated in a 50 °C water bath. The mixture was kept in the ultrasonic bath for 10 min to hydrate the lipid film. The non-encapsulated PTX was then separated from the liposomes by centrifugation at 3000 rpm, 25 °C, for 10 min (Heraeus Multifuge X1R centrifuge, Thermo Fischer Scientific, Waltham, MA, USA). To remove the non-retained ammonium sulfate, the liposomes were kept on dialysis for 24 h against HEPES buffered saline solution (HBS), pH 7.4. The 10 k Da membrane (Sigma-Aldrich, St. Louis, MI, USA) was used for dialysis. The insolubility in water and the high molecular weight makes it difficult for the unencapsulated PTX molecules to escape and pass through the membrane. The concentration of PTX in these liposomes was determined by high-performance liquid chromatography (HPLC).

After determining the PTX concentration, the DXR was incubated with the liposomes to obtain a 1:10 molar ratio of PTX and DXR, respectively. DXR encapsulation was performed by remote loading, conducted by a gradient of transmembrane sulfate. The liposomes containing PTX were kept in contact with DXR concentrated aqueous solution 34 mg/mL for 2 h at 25 °C and then dialyzed against HEPES buffered saline solution (HBS), pH 7.4, for 24 h, to remove unencapsulated DXR [24,28].

#### 2.2.2. Determination of the Content of PTX and DXR in LCFL-PTX/DXR

To determine the encapsulation percentage of the drugs in LCFL-PTX/DXR, freshly prepared samples were analyzed by chromatography and spectrometry methods. For the determination of the PTX concentration after the first dialysis, a centrifugation process is carried out to remove the unencapsulated PTX. After the second dialysis, a new measurement is performed to check if any PTX amount was lost in relation to the first result. The concentration of PTX was measured using the chromatograph composed of a Model 515 pump, a Model 717 Plus autoinjector, and a Model 2996 DAD detector (Waters Instruments, Milford, CT, USA), controlled by Empower software, version 2.0. For analysis, a Hibar column 250-4 LiChrospher 100RP-18, 25 cm × 4 mm, 5 μm (Merck, Darmstadt, Germany) was used. The eluent system consisted of acetonitrile: water (55:45) in a 1.2 mL/min flow. The injection volume was 10 μL and the running time was 8 min. The eluate was detected at a wavelength equal to 227 nm at room temperature [29]. The PTX encapsulation percentage (*EP*) was calculated according to the following equation:

$$EP=\frac{\left[amount\;of\;PTX\;in\;purified\;liposomes\right]}{\left[amount\;of\;PTX\;in\;non\;purified\;liposomes\right]}\times 100$$

UV-VIS spectrophotometry (Thermo evolution 201 UV visible spectrophotometer) was used as the method for quantification of DXR in liposomes at a wavelength equal to 480 nm [30]. The linear regression equation and coefficient of determination (r^2^) were y = 0.01814x + 0.01119 and 0.997, respectively. The accuracy was found to be between 98–104% (n = 3) and samples of blank liposomes showed no absorbance at the wavelength used [24]. Initially, drugs were extracted from the vesicles with isopropyl alcohol at a ratio of 1:2, respectively, and then the preparations were diluted in HEPES buffered saline (HBS) buffer, pH 7. The DXR encapsulation percentage (*EP*) was calculated according to the following equation:

$$EP=\frac{\left[amount\;of\;DXR\;in\;purified\;liposomes\right]}{\left[amount\;of\;DXR\;in\;non\;purified\;liposomes\right]}\times 100$$

#### 2.2.3. Determination of Size, Polydispersity Index, and Zeta Potential of LCFL-PTX/DXR

The diameter of the vesicles and the PI were determined by dynamic light scattering (DLS). The measurements were performed at 25 °C, using a 90° laser incidence angle. The zeta potential (ζ) of the vesicles was determined by DLS associated with electrophoretic mobility. To perform both measurements, 30 µL of liposomes were diluted in 1 mL of HEPES buffered saline (HBS), pH 7, and evaluated on the Zetasizer Nano ZS90 equipment (Malvern, UK).

### 2.3. Preformulation Studies

#### 2.3.1. Freeze-Thawing Studies

The sugars sucrose, trehalose, and glucose were tested independently for their cryoprotective/lyoprotective capacity when added to the external medium of the LCFL-PTX/DXR formulation. The influence of these sugars on the encapsulation content, diameter, ζ, and PI of liposomes after freezing-thawing was evaluated. The choice of concentrations was based on the weight ratio (*w*/*w*) between the cryoprotectants and the phospholipids present in the formulation. In the freeze-thawing study, for each 1 mL of LCFL-PTX/DXR formulation, sucrose, glucose, or trehalose cryoprotectants were added in the weight ratios 2:1 (11.32 mg/mL) and 3:1 (16.98 mg/mL) *w*/*w* in relation to phospholipids. Samples containing trehalose were also tested with concentrations of 5:1 (28.3 mg/mL) and 9:1 (50.94 mg/mL) *w*/*w* in relation to phospholipids. The literature states that disaccharides such as sucrose and trehalose are more effective in protecting liposomes during lyophilization, storage, and rehydration [5,31]. This is because they exhibit reduced hygroscopicity, low chemical reactivity, and higher Tg’ of the maximum frozen-concentrated fraction [5,32,33,34]. However, the Tg’ of dry trehalose is much higher than that of sucrose and remains higher even when this sugar is partially rehydrated. The ability of trehalose to remain vitrified and form dihydrates after water absorption appears to be responsible for this remarkable effect, which makes it superior in preserving liposome structures in the lyophilized form [5,8,31,34,35,36]. After selecting trehalose as a cryoprotectant, we kept the concentrations 5:1 and 9:1 *w*/*w* which are also usual in the literature, since a minimum of 3% by mass of the total solids in the vials is required to obtain a suitable lyophile [37,38,39].

For the intermediate trehalose concentration 5:1 *w*/*w*, a new way of adding cryoprotectants to the formulation was also evaluated: the sugar was added at the time of hydration of the lipid film together with the ammonium sulfate solution.

The liposomes were frozen in immersion in liquid nitrogen for 15 min or slow-frozen by gradual cooling in a freeze-drier (LyoLog Epsilon 2–4 LSCplus, Martin Christ, Osterode Harz, Germany) at a freezing rate of 0.39 °C/min. The freezing point of samples in the freeze-dryer was checked with electrical resistivity sensors. The electrical resistance of a product that is being frozen increases significantly with the change from liquid to solid state, due to reduced ion mobility. This resistivity value is given in percentage, and a value close to 100% indicates that the product is completely solid, that is, frozen [40].

Soon after freezing, all samples were thawed in a water bath at 30 °C for 15 min. Then, each of them was divided in two: one for dialysis against HEPES buffered saline (HBS), pH 7.4, for 24 h, and the other centrifuged at 3000 rpm, 25 °C, for 10 min (Heraeus Multifuge X1R centrifuge, Thermo Fischer Scientific, MA, USA). The dialyzed samples were used to measure the DXR retained in the vesicles. The centrifuged sample was evaluated for PTX retained in the liposomes. The samples were also characterized for vesicle size, PI, and ζ, as described in items 2.3 and 2.4. 

#### 2.3.2. Determination of the Maximum Freeze Concentrated Solute Temperature (Tg’)

The calorimetric analyses were performed using the Mettler Toledo DSC instrument (Schwerzenbach, Switzerland) and the Mettler Toledo MX5 micro-analytical balance (Schwerzenbach, Switzerland). Aliquots of the freshly prepared samples (40 µL) were placed in an aluminum pan, which was closed with the appropriate lid. For the analysis, the equipment was previously stabilized at 25 °C for 5 min. Then, the sample was cooled at 10 °C/min until reaching a temperature of −80 °C. A new stabilization of the equipment was performed at −80 °C for 5 min. Finally, samples were heated to room temperature at 5 °C/min. The data obtained were analyzed by isotherm software of the equipment and for each sample, the glass transition temperature of the maximally frozen concentrated solute (Tg’) was determined.

#### 2.3.3. Determination of Collapse Temperature (Tc)

The Tc determination was performed by freeze-drying microscopy analysis in a microscope coupled to a freeze-drying module, Lyostat 2, model FDCS 196 (Linkam Instruments, Surrey, Redhill, UK), equipped with a liquid nitrogen freezing system (LNP94/2) and programmable temperature controller (TMS94, Linkam). The pressure was monitored by a Pirani valve. The equipment was calibrated with aqueous NaCl solution (eutectic temperature of −21.1 °C). The direct observation of freezing and freeze-drying was performed by a Nikon polarized light microscope, model Elipse E600 (Nikon, Japan). The cooling ramp was from 5 °C/min to −60 °C, followed by 1 min stabilization and a heating ramp of 5 °C/min up to −30 °C, followed by another stabilization for 2 min. Another cooling ramp from 5 °C/min to −60 °C and 1 min stabilization was performed. Finally, heating to 0 °C at a rate of 3 °C/min occurred. The pressure used during the process was 0.1 mTorr. The data were analyzed using the Linksys 32 software, with the collapse temperature determined visually as the one at which the dried sample started to flow (onset) or disrupted (total collapse).

### 2.4. Development of the Freeze-Drying Cycle

Based on the preliminary studies, samples were freeze-dried in a pilot freeze-dryer (LyoLog Epsilon 2–4 LSCplus, Martin Christ, Osterode Harz, Germany). The mass ratios between cryoprotectant and phospholipids chosen were 5:1 and 9:1 *w*/*w*. Samples of 1 mL of the formulations were inserted in amber glass vials with silicone stoppers. Freeze-drying cycle parameters were evaluated, such as temperature, time, and pressure, with the aid of the equipment software, thermocouples, and electrical resistivity sensors. Temperature, ramp rates, and step durations are described and discussed in the results and discussion, respectively. 

### 2.5. Characterization of the Freeze-Dried LCFL-PTX/DXR

#### 2.5.1. Determination of the Residual Moisture Content of the Freeze-Dried LCFL-PTX/DXR

The residual moisture of the freeze-dried samples was determined using a thermogravimetric analyzer (TGA, model TGA-50M, Shimadzu, Kyoto, Japan). The weighing of the samples was carried out in an aluminum pan placed on a microanalytical balance (Mettler Toledo, model MX5, Schwerzenbach, Switzerland). The samples were heated from 25 to 150 °C at 10 °C/min.

#### 2.5.2. Physicochemical Evaluation of Reconstituted Freeze-Dried Products

The freeze-dried liposomes were rehydrated, at room temperature, with 1 mL of ultrapure water, a volume similar to that removed from the sample during freeze-drying. Rehydration was performed gently with the aid of a tube shaker to ensure complete resuspension. The time required for the complete dispersion of the freeze-dried product was determined under direct visual inspection. The content of PTX and DXR was determined and the values were compared to those measured in the colloidal dispersions of the freshly prepared liposomes. For this, the samples were divided into two parts. One of the parts underwent centrifugation to remove PTX that left the vesicles after freeze-drying and the drug was dosed using the HPLC as already described in Section 2.2.2. These samples used for the concentration determination of PTX were also employed to determine the average vesicle size, IP, and ζ as already described in Section 2.2.3. The other part separated from the samples was used for the determination of the DXR concentration. The formulation initially underwent dialysis, for 24 h, against a HEPES buffer to remove the DXR that left the vesicles to the external medium of the dispersion. The dosing was performed by spectrophotometry according to the description cited in Section 2.2.2.

#### 2.5.3. Nanoparticle Tracking Analysis

Nanoparticle tracking analysis (NTA) was performed using a NanoSight NS500 instrument (Salisbury, UK) equipped with a charge-coupled device (CCD) camera that allows visualizing and tracking the Brownian motion of laser-illuminated particles in the aqueous dispersion. The measurements were made at room temperature and each video sequence was captured over 60 s with manual shutter and gain adjustments. The samples were diluted 100,000 times with ultrapure water and then injected into the system (at least three replicates for each formulation).

#### 2.5.4. Cryomicroscopy

The evaluation of the size, morphology, surface, and lamellarity of the vesicles before and after freeze-drying/rehydration was performed using cryomicroscopy. The analyses were performed using a transmission electron microscope JEOL 1400, at 120 kV, at Centro de Microscopia da Universidade Federal de Minas Gerais. The samples were prepared by rapid freezing in liquid ethane in the Vibrobot equipment (ThermoFisher Scientific FEI, Hillsboro, OR, USA). For each sample, a volume of 3 μL was pipetted and deposited on the surface of the carbon film of ultra-thin lacey-carbon (EMS) type copper grids (EMS), previously ionized by oxygen plasma (glow discharge). Immediately before the rapid freezing of the sample by immersion in liquid ethane, the excess sample was removed in an automated way by contacting the grid with absorbent paper in order to leave a film of the solution on the surface of the carbon film. After rapid freezing of the sample, the grid was stored in liquid nitrogen and kept frozen during cryomicroscopy analysis.

## 3. Results

### 3.1. Preparation and Characterization of LCFL-PTX/DXR

As previously shown by Roque and coworkers (2019), LCFL-PTX/DXR formulation presented an adequate diameter, close to 200 nm, low polydispersity, and slightly negative ζ, but close to neutrality. In addition, the encapsulation rates of PTX and DXR were 74.0 ± 2.0, and 89.6 ± 12.3, respectively, which corresponds to a nearly 1:10 drug ratio (Table 1).

### 3.2. Preformulation Studies for Freeze-Dried Formulations

#### 3.2.1. Freeze-Thawing Studies

The physicochemical parameters of the samples submitted to freeze-thawing at a slow cooling rate are presented in Table 1. Freezing, regardless of cryoprotection, decreased PI, mean particle size, and DRX and PTX retention (except with trehalose 5:1, maintaining PTX retention). Upon freezing, PI and ζ values were not significantly affected by the addition of different cryoprotectants or their concentration variation, whereas size changed, but its variation did not correlate with the inputs.

The freezing point values were similar for all samples tested with cryoprotectant to phospholipids equal to 2:1 and 3:1 *w*/*w* ratio. The increase of the concentration of trehalose to 5:1 and 9:1 *w*/*w* decreased the freezing point, as already expected, due to a significant mass increase of solutes.

Freezing liposomes without cryoprotectant incisively affected the retention of both drugs inside the carriers. The retention of PTX in LCFL-PTX/DXR after freezing-thawing was higher for the samples containing glucose (2:1 and 3:1 *w*/*w*) and trehalose (3:1 and 5:1 *w*/*w*) when compared to the sample without cryoprotectant. Different concentrations of sucrose did not alter PTX retention when compared to the sample without cryoprotectant. However, for trehalose, the greatest retention of PTX occurred at 5:1 *w*/*w* ratio, maintaining the pre-freezing values without cryoprotectant.

The retention of DXR after the freezing-thawing process was higher in all samples containing cryoprotectant, except for glucose at a concentration of 2:1 *w*/*w* when compared to the sample without cryoprotectant. Noteworthily, no cryoprotectant stood out in this parameter. The molar ratio between PTX and DXR in the formulations was closer to the original value of 1:10 for frozen-thawed LCFL-PTX/DXR in the presence of trehalose at concentrations of 5:1 and 9:1 *w*/*w*. The cryoprotectant type and concentration change practically did not alter the average size of the vesicles submitted to the freezing step. 

The results related to the physicochemical characterization of the samples that were freeze-thawed under fast freezing mode are presented in Table 2. The standard deviations found for the evaluated parameters were greater than the slow-cooling samples, mainly for drug retention. Among this group, there were no relevant differences for PTX retention, vesicle mean size, ζ, and PI. The highest DXR retentions occurred in samples with trehalose at 5:1 and 9:1 *w*/*w*, whereas the molar ratio between PTX and DXR remained closer to the original value of 1:10 in all samples evaluated. 

Overall, trehalose delivered similar or better outputs than no sugar, glucose, or sucrose and it was chosen as the cryoprotectant for the following studies.

#### 3.2.2. Determination of the Maximally Freeze-Concentrated Solute Temperature (Tg’)

Tg’ of LCFL-PTX/DXR was checked in a standard way (continuous temperature ramp) and also with an annealing step to evaluate possible improvements of the thermal behavior of the formulation. The results are shown in Table 3.

The Tg’ of liposomal dispersions increases with the concentration of some cryoprotective agents [22]. Accordingly, Tg’ of the liposomes containing trehalose 9:1 *w*/*w* in relation to phospholipids was 12.92 °C higher than the formulation of trehalose 5:1 *w*/*w*. It is also possible to observe that the annealing step raised the Tg’ of the formulations a little further for both trehalose concentrations, which favors a faster-drying step during freeze-drying.

#### 3.2.3. Determination of Collapse Temperature (Tc) 

Table 3 also shows the results of the temperature of micro-collapse and total collapse of the LCFL-PTX/DXR samples added with trehalose 5:1 and 9:1 (*w*/*w*), determined by freeze-drying microscopy. For this analysis, the influence of the annealing process on the Tc value was also evaluated. Sugar addition led to a significant reduction of the Tc of the formulations. The inclusion of the annealing step led to the increased collapse and micro-collapse temperatures of liposomal formulations containing both concentrations of trehalose (5:1 and 9:1). These temperatures were higher with the use of trehalose 9:1 *w*/*w*.

In conclusion, LCFL-PTX/DXR plus trehalose at the highest concentration combined with annealing during freezing presented the highest Tc among the samples containing this sugar.

### 3.3. Development of Lyophilization Cycles 

**Figure 1:**
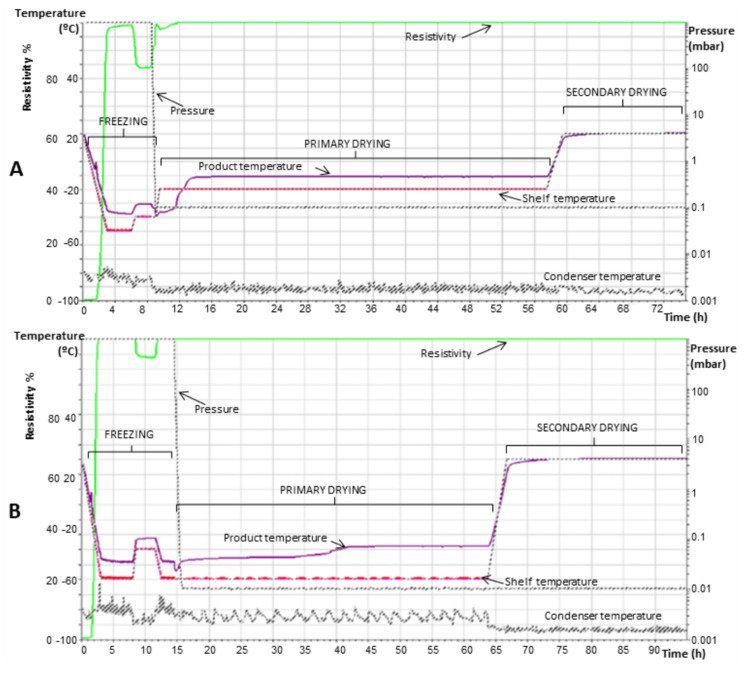
Graph of the complete freeze-drying process using cycles 1 (**A**) and 2 (**B**). Graph of freeze-drying of LCFL-PTX/DXR added of trehalose 9:1 *w*/*w* in relation to the phospholipids. Cycle 1 lasted 74 h and cycle 2 lasted 90 h.

Based on the pre-formulation studies, lyophilization cycles were defined. The process was monitored in real-time for shelf temperature, product temperature, resistivity, and chamber pressure. For freezing, the shelf temperature was set to −50 °C to freeze LCFL-PTX/DXR added with trehalose 5:1 and 9:1 *w*/*w* (cycle 1). This temperature is below the freezing temperatures of all samples in the freeze-thawing test, as well as below the Tg’ determined for most samples. The freezing time of 6 h was chosen according to the freeze-thawing tests, observing the electrical resistivity always above 90% (cycle 1 without annealing). Other samples passed through annealing at a temperature between the freezing point and Tg’ [13,41]. Primary drying was set up to occur at −20 °C, which is above the Tcs found for the liposomes. Table 4 shows all the freeze-drying parameters used in cycle 1 with annealing and Figure 1A shows the behavior of LCFL-PTX/DXR with threalose 9:1 *w*/*w* and process parameters over time.

In order to follow the conventional line for primary drying, another lyophilization cycle, called cycle 2, was also evaluated with the formulation with the best results after freeze-drying under cycle 1 parameters (LCFL-PTX/DXR added with trehalose 9:1 *w*/*w*). Table 4 displays the freeze-drying parameters used in cycle 2. In this second cycle, the main objective would be to reduce the shelf temperature during primary drying to −60 °C as well as to reduce the pressure to 0.01 mBarr, in order to achieve a more effective drying, at temperatures significantly below Tc. For this, the shelf temperature during freezing also had to be reduced to −60 °C. Figure 1B shows the graph of the freeze-drying process of LCFL-PTX/DXR added with trehalose 9:1 *w*/*w* for cycle 2 with annealing.

### 3.4. Characterization of the Lyophilized and Rehydrated LCFL-PTX/DXR

#### 3.4.1. Macroscopic Evaluation, Moisture Determination, and Reconstitution Times of Freeze-Dried LCFL-PTX/DXR

**Figure 2:**
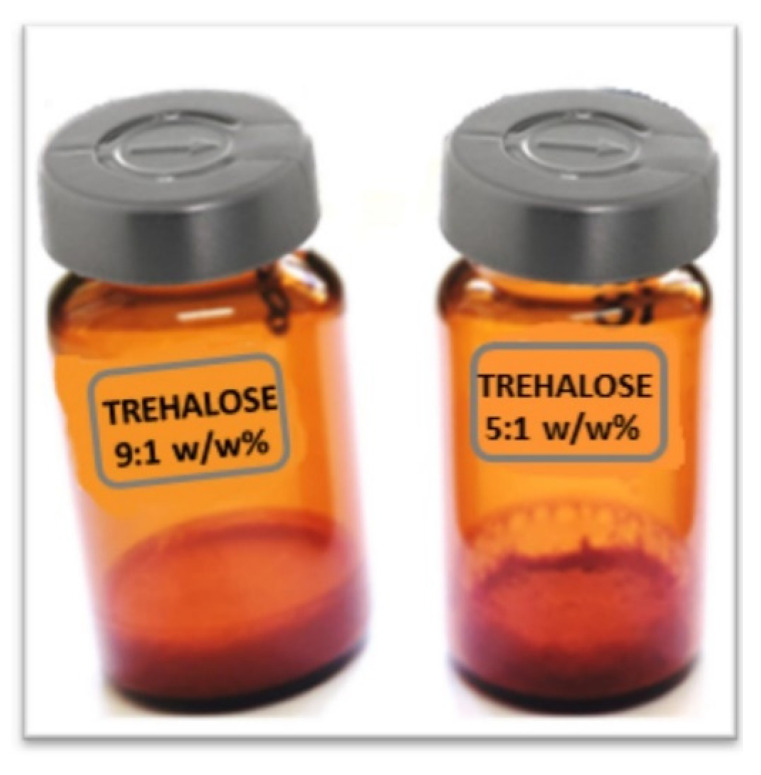
Freeze-dried LCFL-PTX/DXR resulting from freeze-drying cycles 1 and 2. Freeze-dried LCFL-PTX/DXR containing trehalose 9:1 *w*/*w* maintained the structure and volume during the freeze-drying process forming an elegant cake. Freeze-dried LCFL-PTX/DXR containing trehalose 5:1 *w*/*w* suffered shrink.

The bulk structure of LCFL-PTX/DXR samples varied according to the concentration of lyo/cryoprotectant. For samples with trehalose in the proportion 5:1 *w*/*w* of sugar and phospholipid, the freeze-dried products collapsed and shrunk, regardless of the type of freeze-drying cycle. In turn, all freeze-dried LCFL-PTX/DXR samples with trehalose 9:1 *w*/*w* resulted in an elegant cake (Figure 2). All dry products were easy to resuspend, not exceeding 30 s for reconstitution. The samples added with trehalose 5:1 and 9:1 *w*/*w* in the cycle 1 (with annealing) presented 6.8 ± 0.9 and 6.9 ± 1.3% of moisture content, whereas the one from cycle 2 (9:1 *w*/*w*) reached 5.0 ± 1.1%.

#### 3.4.2. Physicochemical Analysis of Freeze-Dried LCFL-PTX/DXR after Reconstitution

The addition of cryoprotectant increased the retention of PTX (Table 5) up to eight times, with the increase in trehalose resulting in better retention. With regard to process changes, the annealing step increased PTX retention for both samples, with statistical difference only for the higher percentage of trehalose. Neither the addition of trehalose nor the annealing process had a statistical difference in DXR retention when compared to no cryoprotectant. 

The variations in the retention of PTX and DXR in face of different concentrations of cryoprotectant and the exposure to different processes resulted in the oscillation of the molar ratios between the two drugs inside the carrier. The polydispersion index of the liposomes remained close to 0.3 and did not differ between samples and processes. The size was significantly smaller for the cycle 2 sample.

#### 3.4.3. Nanoparticle Tracking Analysis

The NTA allows the simultaneous determination of the size and concentration of nanoparticles in a sample [42,43,44,45]. The NTA performed with the newly prepared formulation and the formulation after freeze-drying (cycle 2) with trehalose 9:1 *w*/*w* showed no significant difference in the number of liposomal vesicles. In addition, there was no significant difference between the average vesicle sizes and among the size distributions (D10, D50, and D90) (Table 6).

#### 3.4.4. Cryomicroscopy

**Figure 3:**
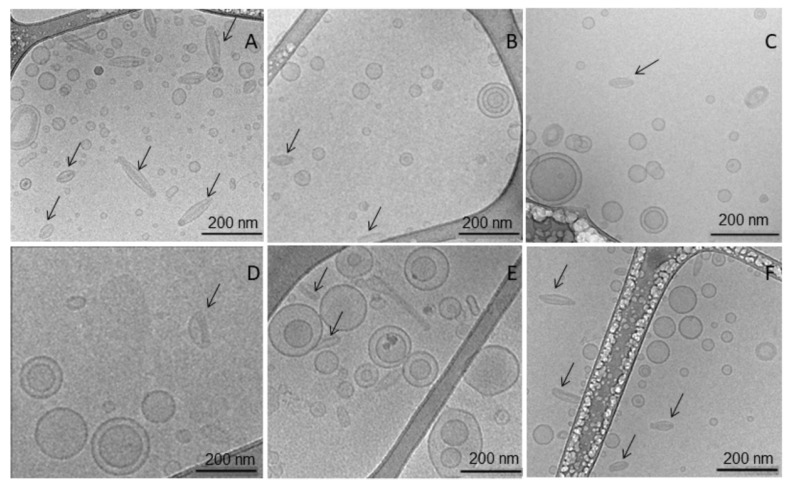
Cryomicroscopy images of the LCFL-PTX/DXR after freezing-thawing and freeze-drying processes. (**A**): Freshly prepared LCFL-PTX/DXR. (**B**,**C**): LCFL-PTX/DXR with trehalose 5:1 *w*/*w* and 9:1 *w*/*w* respectively, after freezing and thawing. (**D**,**E**): LCFL-PTX/DXR with trehalose 5:1 *w*/*w* and 9:1 *w*/*w* after fast freezing using N_2(l)_. (**F**): LCFL-PTX/DXR sample containing trehalose 9:1 *w*/*w* after complete freeze-drying with cycle 2.

Cryomicroscopy allowed investigation of the morphological characteristics of LCFL-PTX/DXR that went through the freeze-thawing and freeze-drying process. Images of cryomicroscopy of freshly prepared LCFL-PTX/DXR were the control group (Figure 3A).

The samples that were frozen and thawed were LCFL-PTX/DXR containing trehalose 5:1 *w*/*w* (Figure 3B) and trehalose 9:1 *w*/*w* (Figure 3C). Additionally, verified was the kind of interference that fast freezing using N_2(l)_ would have on the latter mentioned samples (Figure 3D,E). Finally, cryomicroscopy of LCFL-PTX/DXR samples containing trehalose 9:1 *w*/*w* was performed after complete lyophilization cycle 2 (Figure 3F).

In general, cryomicroscopy images revealed that the vesicular population is heterodisperse in terms of shape and size, however without aggregation or fusion. Most of them have a spherical shape; in some cases, the vesicles are elongated, being mostly unilamellar. In the first image (Figure 3A), referring to the freshly prepared sample, the amount of spherical and prolate ellipsoidal shape vesicles is greater. These vesicles present structures in the form of an electron-dense needle, supposedly representing stacked doxorubicin molecules, indicated by arrows. In Figure 3B, these structures are also present, although in smaller quantities, appearing as larger and less contrasting spherical vesicles.

## 4. Discussion

### 4.1. Preformulation and Freeze-Drying Process Design

#### 4.1.1. Freeze-Thawing

The freeze-thawing study evaluates drug stability in the frozen state in each formulation. In our case, freezing and freeze-drying led to drug leakage of liposomes without cryoprotectants. It is known that the presence of PEG molecules on the surface of the liposomes inhibits the formation of ice crystals during freezing. It interacts with water molecules of the hydration layer of the vesicles and, consequently, reduces the local ice nucleation [5,34]. Reports show that pegylation generally does not protect the liposomes during the freeze-drying process. Thus, these liposomal dispersions still require cryoprotectants to avoid aggregation or fusion of vesicles in freeze-drying [5]. 

There is no single generally accepted theory regarding the action of cryoprotectants, being that these compounds exert their action via one or more of the following mechanisms. The water replacement theory attributes the stabilization effect of protectors to their ability to replace the bound water around the bilayers through specific interactions with the polar region of the lipid head group at low hydrations. In the vitrification theory, a highly viscous matrix is formed around the liposome which reduces the mobility during the freeze-drying process [8,46]. Kosmotropic effects, the less common theory, establishes that cryoprotectants interact with water and disrupt the normal structure. The damage during freeze-drying is prevented due to the reduction of water content at the membrane interface [11]. Despite the theories, deeper studies must be undertaken to understand their protective effect on freeze-dried liposomes. Disaccharides such as trehalose reduced the osmotic stress, stabilized the liposome, and eventually protected the integrity of the liposomes. When liposomes are frozen, the excipient keeps the size of the liposomes constant, while also reducing the osmotic gradient caused by cryo-concentration. On the contrary, the excipient stabilizes the lipid bilayers in the liposomes’ outer compartment, limiting changes in their physical characteristics and eventual drug leakage [9].

Despite the similar results between LCFL-PTX/DXR formulations with different cryoprotectants, some advantages led to the choice of trehalose in concentrations 5:1 and 9:1 *w*/*w*: the highest values of DXR retentions (fast freezing), full PTX retention (5:1, slow freezing) or little loss (9:1, quick freezing). For slow cooling, trehalose also maintained a molar ratio between the drugs close to the original value of 1:10.

Samples of LCFL-PTX/DXR that underwent quick freezing showed a behavior very similar to those that underwent slow freezing, which indicates that the cooling rates tested had little influence on the content and structure of the liposomes. In view of this and considering that a process entirely carried out inside the freeze dryer facilitates the production, the slow freezing of LCFL-PTX/DXR in a freeze dryer was used for the following steps.

Reports describe those disaccharides, such as sucrose and trehalose, are more effective in protecting liposomes during freeze-drying, storage, and rehydration [5,31]. The reason is that they exhibit reduced hygroscopicity, low chemical reactivity, and higher Tg’ of the maximally concentrated fraction by freezing than other saccharides [5,32,33,34]. In addition, Tg value for dry trehalose is higher than that for sucrose and remains higher even when this sugar is partially rehydrated. The ability of trehalose to remain vitrified and form dihydrates after water absorption seems to be responsible for this remarkable effect, better stabilizing biomaterials, which makes it superior for preserving the structures of liposomes [5,8,31,34,35,36]. After selecting trehalose as a cryoprotectant, it was considered that a minimum amount of 3% of total solids in the flasks was necessary for a good cake structure. Thus, concentrations 5:1 and 9:1 *w*/*w* of trehalose in relation to total phospholipids were used. These ratio choices were based on previous reports [37,38,39].

#### 4.1.2. Tg’ and Tc of LCFL-PTX/DXR and Development of Complete Freeze-Drying Cycles

Among the samples containing trehalose 5:1 and 9:1 *w*/*w*, the one with the highest concentration exhibited higher Tg’ values. The insertion of higher concentrations of trehalose between the lipid chains can cause the reduction of intermolecular interactions, with a consequent increase in the molecular mobility of these chains. This reduces the energy level needed to give them mobility, thus reducing the Tg’ value of the lipid. The Tc values followed the same behavior, being higher for the formulation containing trehalose 9:1 *w*/*w* [5,8]. Thus, this cryoprotectant a higher percentage is preferred since it allows the use of higher drying temperatures.

This increase in Tc due to an increase in trehalose concentration was already reported, both for binary solutions (water + trehalose) and for more complex solutions containing cell culture media such as DMEM and RPMI. In the latter, the effects are more significant. For example, in solutions containing DMEM, increasing the concentration of trehalose from 0.05 M to 0.8 M causes an increase in Tc by up to 30 °C [47]. 

Conventionally, the temperature of the primary drying should be adjusted from 5 to 10 °C below the Tc or Tg’ of the formulation [5,8]. However, many studies have shown that liposomal dispersions can be dried primarily at temperatures higher than their Tg’ without changing the physical and chemical characteristics of the products. The proposed mechanism applicable to liposomes is that when drying at a temperature above Tg’, the high sublimation rate is fast enough to complete the drying of the product before significant structural changes in the lipid bilayer [5].

In an attempt to enhance the sublimation step and speed up the drying process, the shelf temperature was set at −20 °C in cycle 1 of lyophilization. The determined pressure of 0.1 mBarr, when evaluated on the curve of water vapor pressure and temperature, is equivalent to a temperature of −42 °C. Therefore, even if the shelf temperature is above the Tg’ and Tc values, the temperature of the product would still be lower. The reduced pressure has a greater influence on maintaining the low temperature of the sample than the temperature of the shelf itself. 

To follow the conventional line for primary drying, another freeze-drying cycle (cycle 2) was evaluated with the most promising sample found in cycle 1 (LCFL-PTX/DXR added trehalose 9:1 *w*/*w*). In cycle 2 of freeze-drying, the main objective was to reduce the shelf temperature and chamber pressure to achieve effective drying at temperatures below Tc. For this, the shelf temperature during the freezing step was also reduced and the duration of some process steps was adjusted. In the end, these changes increased the duration of this cycle by 16 h when compared to the previous one.

### 4.2. Characterization of the Freeze-Dried Product

#### Evaluation of Chemical, Physicochemical, and Morphological Characteristics of Freeze-Dried LCFL-PTX/DXR 

Desired characteristics for freeze-dried products include their elegant appearance and short reconstitution time. In addition, the reconstituted dispersions must preserve all the characteristics of the original formulation, which guarantees the protection of liposomes during the freeze-drying process, such as particle size, ζ, and encapsulation percentage [5]. LCFL-PTX/DXR containing trehalose 9:1 *w*/*w* and freeze-dried under cycle 2 resulted in an elegant cake with no collapse signs and a short reconstitution time. After rehydration, the mean size and charge of the vesicles remained closer to the initial liquid formulation in the absence of the cryoprotectant, whereas the polydispersity index tended to have a higher value. Although trehalose 5:1 *w*/*w* did not prevent matrix collapse, it did preserve the physical outputs of the resuspended liposomes.

Unfortunately, drug retention values of drugs in our study decreased upon drying, especially the DXR one, to at least half the initial values. The leakage of hydrophilic molecules from liposomes is frequently reported in the literature, whereas the increments in sugar ratio did not promote drug retention at desirable levels. Non-stealth liposomes loaded with carboxifluorescein leaked 80% of their content after freeze-drying with sucrose up to 10:1 sugar:lipid ratio. However, particle size and PDI were maintained, showing prevention of vesicle fusion and aggregation [48]. Stealth liposomes loaded with doxorubicin also presented leakage at room temperature, which was independent of the studied PEG concentrations. For the hydrophobic molecule Simvastatin, PEG concentration was positively correlated with drug retention in stealth liposomes, whereas cholesterol increments were negatively correlated with encapsulation rates [49].

The escape of encapsulated drugs is one of the factors that limit the development of commercial freeze-dried liposomal products [49]. The solubility and partition characteristics of the drug determine whether it is easy to permeate the bilayer and release from liposomes or not. A highly lipophilic drug such as PTX shows easier retention in liposomes, due to its low water solubility after rehydration, when compared to amphiphilic and highly hydrophilic drugs, such as DXR. Nonetheless, there are reports showing acceptable retention values after freeze-drying of liposomes containing drugs with different properties [8]. 

The heterogeneity between the vesicles observed by cryomicroscopy complies with the PI and NTA values of the LCFL-PTX/DXR samples. PIs were close to or higher than 0.3, indicating a heterodisperse population [50]. However, the diameter of the vesicles was equal to 200–250 nm, an adequate value for intravenous administration in antineoplastic therapies. In particular, LCFL-PTX/DX with trehalose 9:1 *w*/*w* and freeze-dried with cycle 2 presented a significant minor value (approximately 200 nm) [51,52].

Reduced moisture content is desired for greater stability of solid medicines. Yet some formulations need a residual water amount to remain viable. The most important factors that influence the residual moisture content are the type and concentration of the cryoprotectant and the parameters of the freeze-drying process [49]. In our case, the longer drying time of cycle 2 with the lower pressure enabled a higher drying.

## 5. Conclusions

Our work explored freezing/drying parameters and sugar addition in long-circulating and fusogenic liposomes loaded with PTX and DXR. Thehalose did protect the cargo upon freezing, besides the other physicochemical outputs. Although frozen medicines do not provide the desired storage temperature, they can provide adequate shelf life and are not impeditive for drugs used in hospitals. In addition, this formulation would be ready to use upon thawing, excluding the need for complex manipulation before use, such as that needed for many chemotherapies in the market. Therefore, the frozen formulation developed here could be an option to allow clinical studies and manufacturing scale-up.

Regarding freeze-drying, trehalose promoted a solid cake, resulting in resuspended vesicles with adequate size, PI, and ζ. Interestingly, the accelerated drying did not affect the results, which promoted a shorter cycle. However, the release of both encapsulated drugs, mainly DXR, occurred during freeze-drying. It would be necessary to perform new biological studies addressing side effects and antitumor synergy in order to confirm that the freeze-dried formulation resembles the original formulation. 

## Figures and Tables

**Table 1:** Chemical and physicochemical characterization of LCFL-PTX/DXR after freeze-thawing using slow cooling rate *.

Ratio Cryoprotectant (*w*/*w*)	Cryoprotectant	PTX Retention (%)	DXR Retention (%)	MR (DXR/PTX)	Size (nm)	PI	ζ (mV)	FP (°C)
2:1	Sucrose	63.2 ± 1.0 ^abd^	65.5 ± 1.1 ^abc^	1:13	175.9 ± 1.9 ^ac^	0.24 ± 0.01 ^a^	−3.66 ± 0.66 ^a^	−38.1
	Trehalose	59.0 ± 0.6 ^ad^	68.4 ± 1.3 ^ac^	1:15	172.3 ± 2.5 ^ab^	0.23 ± 0.01 ^a^	−4.51 ± 0.29 ^a^	−38.0
	Glucose	64.3 ± 2.2 ^ab^	61.5 ± 2.2 ^bcd^	1:12	178.8 ± 5.4 ^ac^	0.26± 0.05 ^a^	−4.49 ± 0.12 ^a^	−38.4
3:1	Sucrose	60.4 ± 3.0 ^abd^	67.3 ± 2.4 ^abc^	1:14	169.2 ± 5.4 ^ab^	0.23 ± 0.01 ^a^	−4.91 ± 0.95 ^a^	−36.3
	Trehalose	65.9 ± 1.3 ^b^	68.9 ± 2.8 ^ac^	1:14	177.3 ± 3.8 ^ac^	0.23 ± 0.01 ^a^	−4.02 ± 0.31 ^a^	−37.5
	Glucose	64.1 ± 2.7 ^ab^	69.1 ± 2.3 ^ac^	1:13	175.2 ± 1.5 ^ac^	0.21 ± 0.03 ^a^	−3.87 ± 0.60 ^a^	−39.3
5:1	Trehalose	72.9 ± 1.9 ^c^	69.7 ± 2.0 ^a^	1:8	161.1 ± 1.3 ^b^	0.20 ± 0.01 ^a^	−5.30 ± 1.04 ^a^	−32.9
9:1	Trehalose	62.3 ± 1.6 ^abd^	62.7 ± 4.0 ^c^	1:9	185.9 ± 6.6 ^c^	0.23 ± 0.02 ^a^	−5.96 ± 1.97 ^a^	−31.8
Without cryoprotectant		57.6 ± 2.4 ^d^	55.3 ± 2.4 ^d^	1:9	186.2 ± 3.3 ^c^	0.25 ± 0.01 ^a^	−4.88 ± 0.62 ^a^	−37.9
Without cryoprotectant, before freezing **		74.0 ± 2.0	89.6 ± 12.3	1:10	211.8 ± 16.3	0.29 ± 0.02	−7.39 ± 2.20	-

* The cooling rate was equal to 0.39 °C/min. PTX: paclitaxel; DXR: doxorubicin; MR: molar ratio between PTX and DXR; PI: polydispersity index; ζ: zeta potential; FP: freezing point. The results are presented as mean ± standard deviation from the mean (n = 3). The data were evaluated by the ANOVA One-way test followed by Tukey’s post-test at a significance level of 5%. Letters (a–d) are a visual representation to indicate the statistical significance between the treatments. Equal letters indicate treatments that are statistically equal to each other (*p* > 0.05). ** values for reference, no statistical comparison.

**Table 2:** Physicochemical characterization of LCFL-PTX/DXR after freeze-thawing using quick cooling rate.

Ratio Cryoprotectant (*w*/*w*)	Cryoprotectant	PTX Retention (%)	DXR Retention (%)	MR	Size (nm)	PI	ζ (mV)
2:1	Sucrose	67.9 ± 10.2 ^a^	68.4 ± 1.3 ^ab^	1:11	177.5 ± 3.2 ^a^	0.27 ± 0.03 ^a^	−3.21 ± 0.47 ^a^
	Trehalose	72.0 ± 6.1 ^a^	70.0 ± 2.1 ^ab^	1:11	177.8 ± 1.7 ^a^	0.28 ± 0.04 ^a^	−3.67 ± 1.37 ^ab^
	Glucose	70.0 ± 8.1 ^a^	64.1 ± 1.4 ^abc^	1:10	176.1 ± 3.0 ^a^	0.28 ± 0.01 ^a^	−3.34 ± 1.37 ^a^
3:1	Sucrose	68.2 ± 10.6 ^a^	68.3 ± 0.9 ^ab^	1:11	177.0 ± 4.2 ^a^	0.29 ± 0.06 ^a^	−3.67 ± 1.67 ^ab^
	Trehalose	69.4 ± 2.5 ^a^	70.1 ± 2.4 ^ab^	1:12	178.4 ± 2.5 ^a^	0.25 ± 0.03 ^a^	−3.23 ± 1.10 ^a^
	Glucose	71.4 ± 1.9 ^a^	62.4 ± 0.7 ^bc^	1:10	179.2 ± 1.6 ^a^	0.27 ± 0.06 ^a^	−3.33 ± 0.95 ^a^
5:1	Trehalose	63.5 ± 2.2 ^a^	72.3 ± 4.4 ^a^	1:9	194.6 ± 2.3 ^b^	0.28 ± 0.05 ^a^	−6.55 ± 0.44 ^b^
9:1	Trehalose	65.0 ± 1.6 ^a^	73.4 ± 4.8 ^a^	1:11	183.8 ± 0.3 ^a^	0.23 ± 0.01 ^a^	−5.67 ± 0.27 ^ab^
Without cryoprotectant		58.5 ± 4.0 ^a^	57.4 ± 6.7 ^c^	1:8	179.5 ± 3.9 ^a^	0.27 ± 0.03 ^a^	−5.65 ± 0.49 ^ab^
Without cryoprotectant, before freezing **		74.0 ± 2.0%	89.6 ± 12.3%	1:10	211.8 ± 16.3	0.29 ± 0.02	−7.39 ± 2.20

PTX: paclitaxel; DXR: doxorubicin; MR: molar ratio between PTX and DXR; PI: polydispersity index; ζ: zeta potential; FP: freezing-point. The results are presented as mean ± standard deviation from the mean (n = 3). The data were evaluated with the ANOVA One-way test followed by Tukey’s post-test at a significance level of 5%. Different letters represent statistical differences. ** values for reference, no statistical comparison.

**Table 3:** Determination of the Tg’ and collapse temperature of LCFL-PTX/DXR at different trehalose concentrations and freezing modes.

	Standard Freezing Mode (°C)			Freezing with Annealing (°C)	
		Trehalose/Phospholipids Ratio			
	Ø	5:1	9:1	5:1	9:1
Glass Transition (Tg’)	−24.14	−53.30	−40.38	−50.98	−39.70
Microcolapse	−28.10	−51.50	−50.40	−47.10	−45.50
Collapse	−26.00	−50.00	−47.60	−43.80	−42.20

Standard: freezing without the annealing step; Annealing: freezing with annealing step. Ø = without thehalose. Trehalose glass transition (reference) = −27.06 °C.

**Table 4:** Parameters used in cycles 1 and 2 of freeze-drying of LCFL-PTX/DXR with annealing step *.

**CYCLE 1**				
**Step**	**Ramp** **(°C/min)**	**Shelf Temperature** **(°C)**	**Pressure** **(mbar)**	**Hold Time (h)**
Freezing 1	0.39	−50	Ambient	3
Annealing	0.34	−40	Ambient	2
Primary Drying	0.34	−20	0.1	48
Secondary Drying	0.34	20	0.1	15
**CYCLE 2**				
**Step**	**Ramp** **(°C/min)**	**Shelf Temperature** **(°C)**	**Pressure** **(mbar)**	**Hold Time (h)**
Freezing 1	0.45	−60	Ambient	5
Annealing	0.67	−40	Ambient	2
Freezing 2	0.34	−60	Ambient	2
Primary Drying	0.34	−60	0.01	48
Secondary Drying	0.39	20	0.01	27

* Total process time in cycle 1 was 74 h and in 2 was 90 h.

**Table 5:** Physicochemical characterization after freeze-drying/reconstitution of LCFL-PTX/DXR formulations.

Cycle		Retention PTX (%)	Retention DXR (%)	MR	Size (nm)	PI	ζ (mV)
1	Without cryoprotectant	7.1 ± 1.5 ^a^	23.5 ± 15.5 ^a^	1:37	326.9 ± 24.8 ^a^	0.28 ± 0.03 ^a^	−2.38 ± 0.15 ^a^
	T5:1 Standard	26.6 ± 2.2 ^b^	27.3 ± 2.7 ^a^	1:9	262.4 ± 2.6 ^a^	0.30 ± 0.01 ^a^	−2.29 ± 0.29 ^a^
	T5:1 Annealing	31.2 ± 4.3 ^b^	35.2 ± 4.8 ^a^	1:10	244.8 ± 9.4 ^ab^	0.32 ± 0.02 ^a^	−2.45 ± 0.72 ^a^
	T9:1 Standard	44.2 ± 2.6 ^c^	31.3 ± 2.6 ^a^	1:7	213.4 ± 5.5 ^ab^	0.37 ± 0.02 ^a^	−2.88 ± 0.13 ^a^
	T9:1 Annealing	56.9 ± 2.5 ^d^	34.9 ± 2.8 ^a^	1:5	240.1 ± 3.2 ^ab^	0.38 ± 0.01 ^a^	−6.46 ± 0.92 ^b^
2	T9:1 Annealing	51.8 ± 4.3 ^cd^	24.6 ± 5.2 ^a^	1:4	204.7 ± 8.3 ^b^	0.36 ± 0.04 ^a^	−2.60 ± 0.13 ^a^

PTX: paclitaxel; DXR: doxorubicin; T5:1= trehalose to phospholipid ratio (5:1 *w*/*w*); T9:1 = trehalose to phospholipid ratio (9:1 *w*/*w*); Standard: freezing without the annealing step; annealing: freezing with annealing step; MR = molar ratio between drugs PTX and DXR; PI = polydispersity index; ζ = zeta potential. The drug retention rates, size, PI, and ζ are presented as mean ± standard deviation from the mean. The PTX and DXR retention percentage and ζ variables were evaluated by the One-way ANOVA test followed by Tukey’s post-test at a significance level of 5%. For the analysis of the size and PI variables, the Kruskal-Wallis test was used followed by the Dunn’s post-test, also at a significance level of 5%. Distinct letters represent statistical differences.

**Table 6:** Characterization of LCFL-PTX/DXR by Nanoparticle Tracking Analysis (NTA).

Analysed Parameters	Before Freeze-Drying	After Freeze-Drying/Rehydration
D10 (nm)	89.6 ± 5.6	90.0 ± 7.6
D50 (nm)	121.4 ± 9.0	123.7 ± 16.4
D90 (nm)	205.1 ± 6.5	217.0 ± 22.4
Average size (nm)	105.1 ± 15.8	109.2 ± 11.9
Particle concentration/mL	2.5 × 10^13^ ± 1.0 × 10^13^	2.2 × 10^13^ ± 7.4 × 10^12^

D10: the portion of particles with a diameter smaller than this value is equal to 10%. D50: the portion of particles with diameter smaller than this value is 50%. D90: the portion of particles with a diameter below this value is 90%. The results are presented as mean ± standard deviation from the mean (n = 3). The data were evaluated with the T-Student test at a significance level of 5%.

## Data Availability

Not applicable.
